# Antibiotic treatment to prevent pediatric acute otitis media infectious complications: A meta-analysis

**DOI:** 10.1371/journal.pone.0304742

**Published:** 2024-06-17

**Authors:** Nicole E. Smolinski, Emma J. Djabali, Julie Al-Bahou, Ariel Pomputius, Patrick J. Antonelli, Almut G. Winterstein

**Affiliations:** 1 Department of Pharmaceutical Outcomes and Policy, College of Pharmacy, University of Florida, Gainesville, FL, United States of America; 2 Center for Drug Evaluation and Safety [CoDES], University of Florida, Gainesville, FL, United States of America; 3 College of Medicine, University of Florida, Gainesville, FL, United States of America; 4 College of Pharmacy, University of Florida, Gainesville, FL, United States of America; 5 Health Science Center Libraries, University of Florida, Gainesville, FL, United States of America; 6 Department of Otolaryngology-Head & Neck Surgery, College of Medicine, University of Florida, Gainesville, FL, United States of America; 7 Department of Epidemiology, College of Medicine and College of Public Health and Health Professions, University of Florida, Gainesville, FL, United States of America; Universidade Federal de Sao Paulo/Escola Paulista de Medicina (Unifesp/epm), BRAZIL

## Abstract

**Background:**

Most US children with acute otitis media [AOM] receive prompt antibiotic treatment, though guidelines encourage watchful waiting. Previous systematic reviews of antibiotics versus watchful waiting have focused on symptom resolution and RCTs, limiting the assessment of serious, rare complications. We sought to evaluate these complications by including observational studies.

**Methods:**

RCTs and observational studies that compared antibiotics to placebo or watchful waiting for pediatric clinician diagnosed AOM were identified [PubMed/MEDLINE, Embase, Cochrane Database of Systematic Reviews, Central Register of Controlled Trials, and Web of Science] and reviewed for meta-analysis. Two reviewers independently extracted study characteristics, patient characteristics, and outcomes. We assessed publication bias, study bias with ROBINS-1 and RoB-2 and used random-effects models to assess treatment effects.

**Results:**

24 studies were included. Antibiotics decreased the risk of acute mastoiditis [incidence 0.02%, RR 0.48, 95% CI 0.40–0.59; NNT 5,368]. This protective effect may be underestimated because of misclassification of non-suppurative conditions as AOM. Intracranial complications remained too rare to assess. Antibiotics markedly increased the risk of adverse effects [incidence 10.5%, RR 1.49, 1.27–1.73; NNH 23]. Studies used non-specific criteria for acute mastoiditis, potentially underestimating treatment effects.

**Conclusions:**

Prompt antibiotic therapy reduces the risk for some AOM complications. The NNT to prevent serious, rare complications is high, while the NNH is relatively low. Large-scale population-based observational studies using real-world datasets with validated measures of severe complications are needed to improve understanding of risk factors for serious AOM complications, facilitate more selective antibiotic therapy, and optimize individual outcomes and public health.

## Introduction

Systemic antibiotic therapy has long been central to the treatment of childhood acute otitis media (AOM) in the US; [1] however, recommendations for use have evolved over the last two decades. With the release of guidelines in 2004, followed by a revision in 2013, the American Academy of Pediatrics (AAP) has promoted the option of watchful waiting (i.e., deferring antibiotics with close follow-up based on joint decision-making with the caregiver), particularly in children ≥ 2 years old with uncomplicated AOM [2, 3]. Watchful waiting is supported by a high rate of spontaneous resolution with limited adverse sequelae in children managed without antibiotics [4]. Several systematic reviews have provided compelling evidence that antibiotics yield modest benefits on AOM symptoms and disease duration [5–12].

Antibiotics also carry risk for undesirable outcomes. Gastrointestinal issues are common [11], and some adverse effects can be life threatening [13]. AOM recurrence may be higher in children treated with antibiotics relative to watchful waiting [14]. Even short courses of antibiotics for AOM have been linked to the emergence of antibiotic-resistant pathogens in individual patients [15, 16], an effect that is compounded on the population-level with widespread use [17]. Thus, there are ample reasons to promote judicious antibiotic use in AOM.

Recent research suggests that the vast majority of AOM episodes in the US are still treated with antibiotics immediately after diagnosis [18–21]. Major reasons cited for foregoing watchful waiting include parental reluctance [22, 23], inconvenience of follow-up with patients who do not improve [23], concern for AOM complications [22, 24, 25], and risk of litigation [26]. Provider specialty has shown strong associations as well [18, 27, 28]. Recent work suggests that prescriber preferences may play a greater role in driving antibiotic use than patient factors [18]. These considerations raise questions about the evidence driving the use of antibiotics for AOM in children, especially regarding clinician concerns about an increased risk of AOM complications when using watchful waiting.

Most systematic reviews on the treatment of AOM have included studies with primary outcomes related to symptom and disease resolution. In these efficacy studies, complications, if reported, were generally addressed as secondary outcomes. Of 9 systematic reviews identified, 3 included non-serious complications [9, 10, 29], and only 2 addressed serious infectious complications [9, 29]. These latter reviews were unable to pool data because of a small number of outcomes with inclusion of only RCTs.

Assessments of rare safety outcomes have long relied on population-based observational studies to maximize sample size and longitudinal follow-up. Thus, we conducted a systematic review that amended previous reviews with observational studies to maximize generalizability to real-world populations and the study power needed to assess the risk of AOM complications in children treated with antibiotics relative to watchful waiting.

## Methods

This meta-analysis was conducted following the Preferred Reporting Items for Systematic Reviews and Meta-Analyses (PRISMA) reporting guideline [30].

### Eligibility

Included studies were RCTs and observational studies that compared outcomes among pediatric patients (≤ 20 years old) with an AOM diagnosis who were treated with antibiotics against a control group (i.e., receiving placebo, expectant observation/no antibiotic treatment, watchful waiting). Watchful waiting involves holding antibiotic treatment for 48 to 72 hours, providing symptomatic relief, and instructing the caregivers to return to the provider if symptoms worsen or persist to reassess the need for antibiotics [3, 31]. Observational studies were based on diagnostic (e.g., ICD) and procedure (e.g., CPT) codes; thus, included studies required only the inclusion AOM and outcome codes. Studies did not have to require or report any specific criteria for AOM, beyond a clinician’s summary diagnosis of AOM, or be conducted in a specific healthcare setting but had to report at least 1 outcome of interest.

### Outcomes of interest

Outcomes were selected based on their assumed impact on AOM management decisions and included serious AOM complications (acute mastoiditis (AM), meningitis, epidural or subdural empyema, brain abscess, sepsis, and pneumonia), non-serious infectious complications [tympanic membrane perforation (TMP), contralateral otitis media, AOM recurrence (defined as having another AOM episode after an index episode), recurrent AOM (defined as 3 or more episodes in 6 months OR 4 or more episodes in 12 months), chronic suppurative otitis media (CSOM), and antibiotic adverse effects (e.g., diarrhea, vomiting, rash).

### Search strategy

Databases searched for relevant articles included PubMed/MEDLINE, Embase, Cochrane Database of Systematic Reviews and Central Register of Controlled Trials, and Web of Science, as well as appropriate grey literature sources, including ClinicalTrials.gov. The searches for each database were comprised of both the relevant controlled vocabulary for that database—including MeSH for PubMed/MEDLINE and Emtree for Embase—and keywords synthesized from synonyms for the concepts of Acute Otitis Media, Watchful Waiting, Antibiotic Treatment, Pediatric Patients, and Comparative Studies (S1 Table). There were no limits on publication dates or language. Additional hand-searching of relevant journals and articles was performed by the search team. The final literature search was run on May 30, 2023, and results were uploaded to Covidence for screening.

### Article screening

Initial title and abstract screening were completed by two independent reviewers with a third resolving any conflicts (EJD, NES, JA, SN). Two authors (EJD, NES, JA) independently reviewed the full text of each article against the inclusion criteria with a third resolving any conflicts. We evaluated all articles in English, German, Spanish, Italian, French, Dutch, Swedish, Danish, and Norwegian.

### Risk of bias assessment

Two authors (EJD and NES) independently assessed the methodological quality of the included studies using the RoB 2 tool for RCTs and ROBINS-I for observational studies [32, 33]. We used the Robvis R package for visualization of bias assessments [34].

### Data extraction

Key study features (author, year, study design, study period, data source, age range, participants, intervention, and control descriptions) and outcomes were extracted and cross-checked by three review authors (EJD, NES, JA).

### Statistical analysis

We used a random effects model (due to heterogeneity of participants in the included trials) and reported risk ratios with 95% confidence intervals. For outcomes with significant risk ratios, we calculated the number needed to treat (NNT) as the inverse of the difference between the outcome rate in the untreated group and the antibiotic group. For studies where results were stratified for age subgroups, we pooled within subgroups to derive more granular, age-specific risk estimates. Data analysis and visualization were completed using RevMan 5.4. As a sensitivity analysis, we excluded from pooled risk estimates any studies that were deemed to have a high risk of bias (RoB 2) or serious/critical risk of bias (ROBINS-1). A second sensitivity analysis was performed removing all studies published prior to 2000. This was to account for changes in clinical practice as well as the introduction of the pneumococcal vaccine which decreased the incidence of both AOM and AOM complications [35, 36].

## Results

### Description of the studies

**Fig 1:**
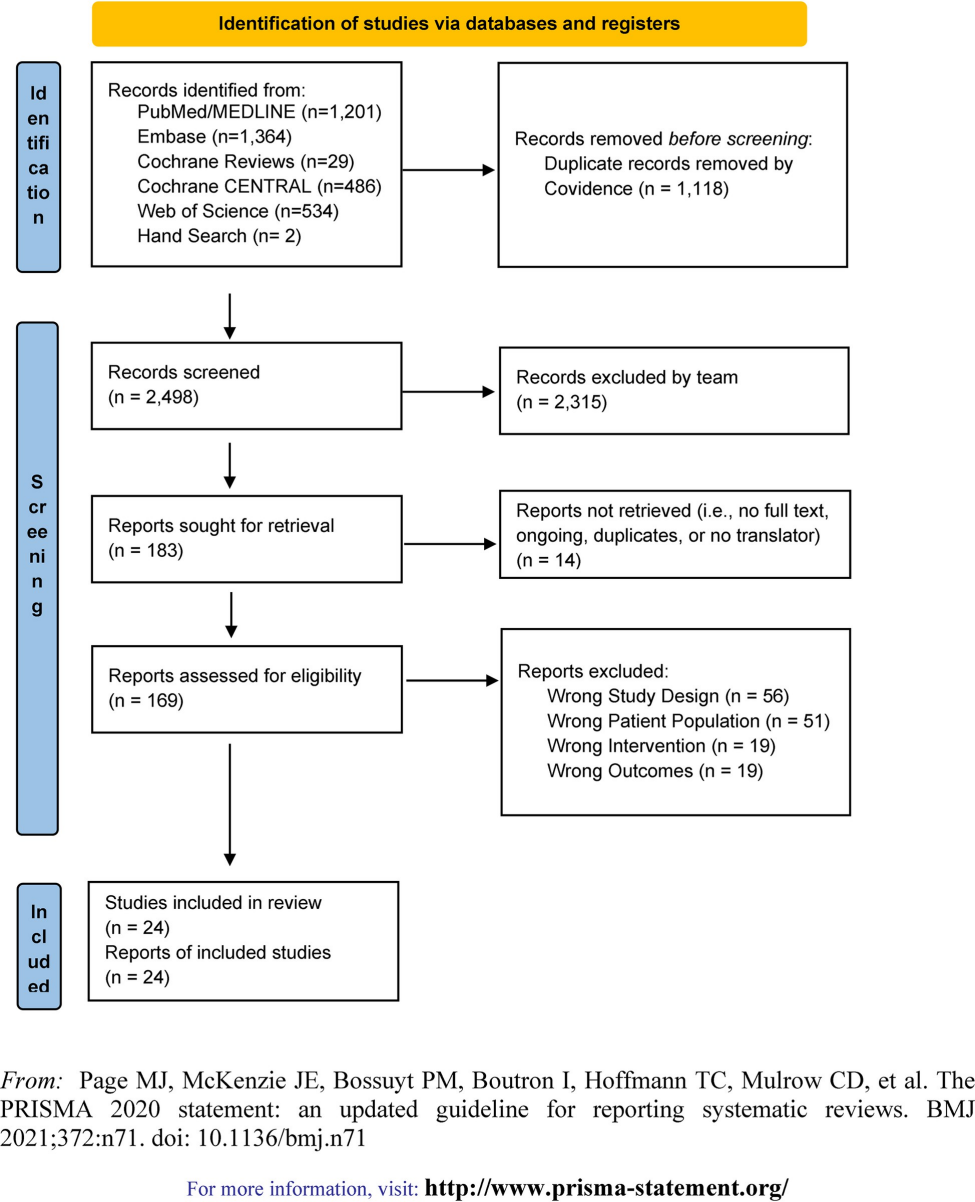
PRISMA flow diagram.

A total of 2,498 articles were identified. After screening, a total of 24 studies met eligibility criteria, including 17 RCTs, 1 RCT follow-up study, and 6 observational studies (Fig 1). Observational study designs included a prospective birth cohort and 5 retrospective cohort studies. One pair of studies included the same cohorts of patients and reported different outcomes in each publication: Tahtinen 2011/Ruohola 2018 [37, 38].

Sample sizes of the RCTs ranged from 84 to 512 (Table 1) [39, 40]. The sample sizes in the observational studies ranged from 214 to 356,906 patients and up to 1,182,272 AOM episodes [22, 41, 42]. Follow-up ranged from 2 weeks to 4 years, though most studies did not exceed 3 months.[43, 44] Twenty-three studies included children only, with ages ranging from 0 to 16 years old. Three studies included both children and adults, with two providing separate data for children, and one only providing data for patients ≤20 years old [22, 24, 25].

**Table 1:** Characteristics of included studies.

First Author and Publication Year	Country/ Setting	AOM Diagnosis Criteria	Study Design	Study period	Participants: intervention/ control	Outcomes extracted	Age range	Follow-up time	Study Groups
**Bezáková 2009**	Netherlands/ General practice	Characteristic ear drum picture or acute otorrhea with one or more symptoms of acute infection	Randomized controlled trial	Feb 1996—May 1998	78/90	AOM recurrence	6–24 months old	46 months	Intervention [Amoxicillin 40 mg/kg/day in three doses for 10 days]
								45 months	Control [Placebo]
**Burke 1991**	United Kingdom/ General practices	Acute earache and at least one abnormal eardrum	Randomized controlled trial	Oct—Apr of 1986–1989	114/118	Antibiotic adverse effects	3–10 years old	1 year	Intervention [Amoxycillin 125 mg three times a day for 7 days]
									Control [Placebo]
**Cars 2017**	Sweden/ Primary, outpatient specialist and inpatient care	ICD 10 Codes: H66.0 Acute suppurative otitis media H66.9 Otitis media, unspecified	Prospective cohort study	Jan 2006 -Jan 2016	331,882/ 66,767 [mastoiditis]*	Mastoiditis, meningitis	0–14 years	1 month	Intervention [List of antibiotics provided]**
					331,897/ 66,768 [meningitis]*				Control [No antibiotic prescription]
**Cushen 2020**	United Kingdom/ Primary care	Read codes	Retrospective cohort study	Jan 1982—Dec 2012	188,769/ 27,680	Mastoiditis, brain abscess	≤20 years old	90 days	Intervention [All oral antibiotics listed in the British National Formulary 64]**
									Control [No antibiotic prescription]
**Damoiseaux 2000**	Netherlands/ General practice	Characteristic ear drum picture or acute otorrhea with one or more symptoms of acute infection	Randomized controlled trial	Feb 1996—May 1998	117/123	Antibiotic adverse effects	6–24 months old	6 weeks	Intervention [Amoxicillin 40 mg/kg/day in divided doses for 10 days]
									Control [Placebo]
**Hoberman 2011**	United States/ Children’s hospital and private pediatric practice	Onset of symptoms that parents rated with a score of at least 3 on the Acute Otitis Media Severity of Symptoms [AOM-SOS] scale, the presence of middle-ear effusion, and moderate or marked bulging of the tympanic membrane	Randomized controlled trial	Nov 2006—Mar 2009	144/147	Tympanic membrane perforation, mastoiditis, AOM recurrence, antibiotic adverse effects	6–23 months old	21 to 25 days	Intervention [Amoxicillin 90 mg/kg/day + Clavulanate 6.4 mg/kg/day for 10 days]
									Control [Placebo]
**Kaleida 1991**	United States/ Children’s hospital and private pediatric practice	Specific symptoms of acute middle ear infection or signs of acute infection or both	Randomized controlled trial	May 1981—Aug 1985	263/273	AOM recurrence	7 months—12 years old	1 year	Intervention [Amoxicillin suspension 40 mg/kg per day in three divided doses for 14 days]
									Control [Placebo]
**Laxdal 1970**	Canada/ Private practice	At least one ear drum showing redness and loss of landmarks	Randomized controlled trial	Jan 1966—Sep 1968	94/48	AOM recurrence	≤ 14 years old	1–33 months [mean 16 months]	Intervention [Either penicillin G or ampicillin, 250 mg/sqm/day in four divided doses for a minimum of 7 days and an average of 10 days]
									Control [Symptomatic therapy]
**Le Saux 2005**	Canada/ Clinics and emergency department	New onset of symptoms with either ear pain or fever, and evidence of middle ear effusion, on the basis of pneumatic otoscopy, and redness or bulging [or both] of the tympanic membrane	Randomized controlled trial	Nov—Mar of 1999–2002	258/254	AOM recurrence, antibiotic adverse effects	6 months—6 years old	3 months	Intervention [Amoxicillin 60 mg/kg/day in 3 divided doses for 10 days]
									Control [Placebo]
Little 2001***	England/ General practice	Acute otalgia and otoscopic evidence of acute inflammation of the ear drum	Randomized controlled trial	NA	151/164	Antibiotic adverse effects	6 months—10 years old	2 weeks	Intervention [Amoxicillin 125 mg three times daily or erythromycin 125 mg four times daily [if penicillin allergy] for 1 week]
									Control [Delayed treatment with antibiotic [72 hrs]]
**McCormick 2005**	United States/ Pediatric clinic	Symptoms of ear infection, otoscopic evidence of AOM, including middle ear effusion	Randomized controlled trial	May 2000—Mar 2003	112/111	AOM recurrence, antibiotic adverse effects	6 months—12 years old	30 days	Intervention [Amoxicillin 90 mg/kg/day, 2 doses per day, maximum of 1500 mg/day, for 10 days]
									Control [Watchful waiting]
**Molder 2016**	Netherlands/ Primary care	International Classification of Primary Care [ICPC] AOM diagnosis codes	Prospective birth cohort study	Dec 2001—Dec 2012	512/336	Recurrent AOM, AOM recurrence	0–2 years old	4 years	Intervention [Oral antibiotics]
									Control [No antibiotic prescription]
**Mygind 1981**	Sweden/ Otologist and general practitioner home visits	Child crying with pain and red/inflamed tympanic membrane	Randomized controlled trial	Nov 1977—Apr 1978	72/77	Tympanic membrane perforations, AOM recurrence, contralateral AOM, antibiotic adverse effects	1–10 years old	3 months	Intervention [Penicillin-V 55 mg/kg/day in divided doses for 7 days]
									Control [Placebo]
									No antibiotic treatment
**Petersen 2007**	United Kingdom/ Primary care	OXMIS16 and Read17 AOM diagnosis codes	Retrospective cohort study	Jul 1991—Jun 2001	309,963/ 46,943	Mastoiditis	0–15 years old	1 month	Intervention [Any antibiotic]
									Control [No antibiotic prescription]
**Roy 2012**	Bangladesh/ Community health workers	Pus/discharge on otoscopy with confirmed diagnosis by ear swab	Retrospective cohort study	NA	182/193	Chronic suppurative otitis media, mastoiditis	0–2 years old	2 years	Intervention [Amoxycillin 50 mg/kg/day for 10 days]
									Control [No antibiotics]
Ruohola 2018***	Finland/ Primary care	1] middle-ear fluid detected by means of pneumatic otoscopic examination, 2] acute inflammatory signs in the tympanic membrane, 3] acute symptoms	Randomized controlled trial	Mar 2006—Dec 2008	161/158	AOM recurrence	6–35 months old	90 days ± 10 days	Intervention [Amoxicillin 40 mg/kg/day + Clavulanate 5.7 mg/kg/day, divided into two daily doses for 7 days]
									Control [Placebo]
**Shahbaznejad 2021**	Iran/ Pediatric infectious diseases clinic	Acute onset of fever, erythema of tympanic membrane, and middle ear effusion	Randomized controlled trial	2016–2018	188/208	Tympanic membrane perforation, AOM recurrence, antibiotic adverse effects, meningitis, mastoiditis	6 months—6 years old	3 months	Intervention [Amoxicillin 80 mg/kg/day divided into two doses for 7–10 days]
									Control [Watchful waiting for 48 hours]
**Spiro 2006**	United States/ Pediatric emergency department	AOM diagnosis at the discretion of the clinician	Randomized controlled trial	Jul 2004—Jul 2005	145/138	Antibiotic adverse effects	6 months—12 years old	40 days	Intervention [Any antibiotic [amoxicillin 80–90 mg/kg/day was prescribed for most patients]]
									Control [Wait-and-see for 48 hours]
Tahtinen 2011***	Finland/ Primary care	1] middle-ear fluid detected by means of pneumatic otoscopic examination, 2] acute inflammatory signs in the tympanic membrane, 3] acute symptoms	Randomized controlled trial	Mar 2006—Dec 2008	161/158	Antibiotic adverse effects, systemic complications contralateral AOM, tympanic membrane perforation, mastoiditis	6–35 months old	8 days	Intervention [Amoxicillin 40 mg/kg/day + Clavulanate 5.7 mg/kg/day, divided into two daily doses for 7 days]
									Control [Placebo]
Tahtinen 2011***	Finland/ Primary care	Same as Tahtinen 2011	Prospective follow-up study from original randomized controlled trial	Mar 2006 –Dec 2008	161/53	Systemic complications	6–35 months old	16 ± 3 days	Intervention [Amoxicillin 40 mg/kg/day + Clavulanate 5.7 mg/kg/day, divided into two daily doses for 7 days]
									Control [Delayed antimicrobial treatment [placebo]
**Tapiainen 2014**	Finland/ Pediatric private practice and pediatric department of hospital	Ear-related symptoms and signs of tympanic membrane inflammation together with MEE detected in pneumatic otoscopy	Randomized controlled trial	Sep 1999 –Jan 2000; Oct 2005 –Dec 2005; and Sep 2009 –Jun 2012	42/42	Mastoiditis, tympanic membrane perforation, antibiotic adverse effects	6 months—15 years old	60 days	Intervention [Amoxicillin-clavulanate 40mg/kg/day for 7 days]
									Control [Placebo]
**Thalin 1985**	Sweden/ Otolaryngology department	Not specified	Randomized controlled trial	Jul 1984—Jun 1985	159/158	Contralateral AOM, AOM recurrence, antibiotic adverse effects	2–15 years old	30 days	Intervention [Phenoxymethyl penicillin 50 mg/kg/day twice daily for 7 days]
									Control [Placebo]
**Thompson 2009**	United Kingdom/ General practice	GPRD medical/product dictionaries AOM diagnosis codes, validated by a pediatrician	Retrospective cohort study	Jan 1990—Dec 2006	792,623/ 389,649*	Mastoiditis	3 months—15 years old	3 months	Intervention [76% received Amoxicillin and 15% Erythromycin]
									Control [No antibiotic treatment]
**van Buchem 1981**	Netherlands/ General practice	Based on history and clinical picture; diffuse redness and/or bulging of the eardrum was taken as decisive	Randomized controlled trial	Jan—May 1979 and Oct—Mar 1980	47/40	Tympanic membrane perforation, mastoiditis	2–12 years old	1–2 years	Intervention [Amoxicillin 250 mg 3x daily for 7 days]
									Control [Placebo]

*Number of AOM episodes as opposed to number of patients

**Treatment duration not provided

***Ruohola 2018 and Tahtinen 2011 include the same cohorts of patients. Tahtinen 2011 and Tahtinen 2011 include the same cohort of patients treated with immediate antibiotics.

NA: not applicable

Amoxicillin was the most used antibiotic. The control group was placebo for 12 of the 19 RCTs. For observational studies, the control groups included no antibiotic prescription/treatment and delayed treatment. Out of the 5 studies with delayed antibiotic therapy as the control group, 1 used a watchful waiting period of 72 hours [43] and 4 used 48 hours [15, 41, 45, 46]. Co-interventions were allowed or encouraged in 18 studies, consisting of symptomatic treatment with analgesics, antipyretics, antihistamines, and decongestants.

Seven studies reported pneumococcal vaccination rates, ranging 1.4–100% [46, 47]. Breastfeeding was reported by 8 studies. Five studies reported the percentage of children who had siblings, ranging from 20 to 69% [14, 44]. Eleven studies reported smoke exposure, ranging 16–39% [46, 48]. Lastly, 12 studies reported attendance at school or daycare, ranging 15–88% [39, 48].

### Assessment of bias

Five RCTs were deemed to have a high risk of bias due to missing information regarding methods for outcome assessment (S1 Fig) [14, 43, 49–51]. Four observation studies were considered to have a moderate risk of bias [22, 25, 42, 44] and 2 of serious risk, due to inadequate adjustment of potential confounders (S2 Fig) [24, 52]. Visual assessment of funnel plots revealed no strong publication bias for any outcome (S3–S5 Figs).

### Heterogeneity

Study results across the evaluated outcomes were homogeneous (I^2^ < 30%) except for AOM recurrence, which had a high heterogeneity (I^2^ = 65%) suggesting systematic differences between these studies rather than just random variation. This is potentially due to the large range in outcome assessment timeframes (6 months to 3.5 years).

### Outcomes

#### Serious infectious complications

**Fig 2:**
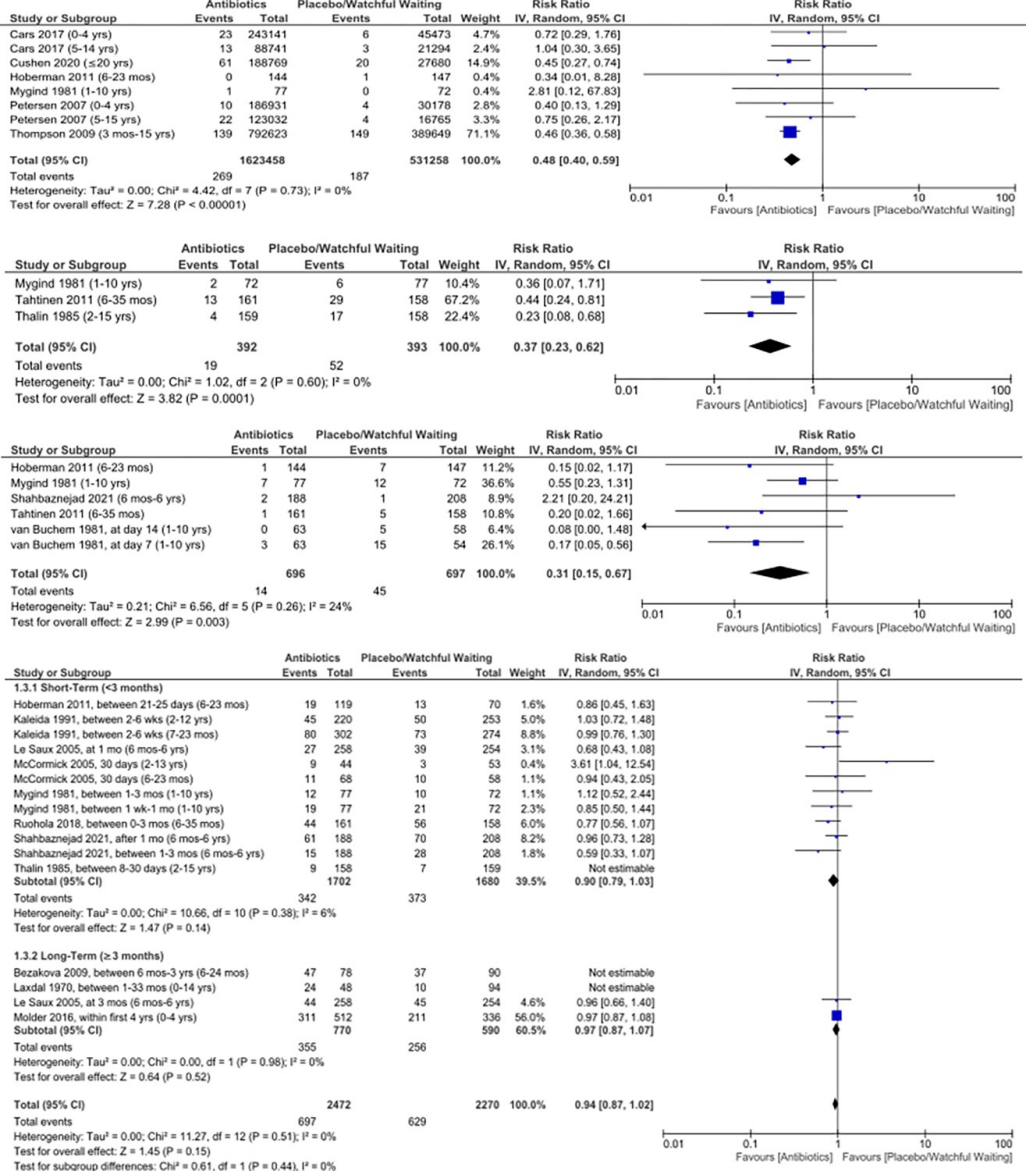
Risk ratios for AOM complications in children treated versus not treated with antibiotics. A: mastoiditis, B: contralateral AOM, C: tympanic membrane perforations, D: AOM recurrence.

Six studies included results for AM as a serious complication of AOM (2 RCTs and 4 observational studies), observing a total of 269 episodes of AM following 1,623,458 treated AOM episodes and 187 episodes of AM following 531,258 untreated AOM episodes [22, 24, 25, 42, 47, 53]. An additional 5 studies reported no AM, out of 630 controls and 610 treated AOM cases [37, 39, 46, 51, 52]. Antibiotics showed a protective effect for acute AM (RR 0.48, 95% CI 0.40–0.59), with an NNT of 5,368 (Fig 2A). The reported time frame allowed for the diagnosis of “AM” was as late as 3 months [25, 53]. Diagnostic criteria varied markedly, with some including chronic mastoiditis and non-specific mastoid disorders [22, 42] and others not defining the criteria [25, 47, 53]. Lacking were combined requirements of an AM diagnosis code and admission for treatment with parenteral antibiotics and / or a cortical mastoidectomy.

Several studies aimed to assess intracranial complications, but only 2 observational studies had sufficient sample size to observe these outcomes [24, 25]. Cars 2017 identified one case of meningitis in 331,897 treated AOM episodes and three in the untreated group (n = 66,768) within 30 days of an AOM episode. The calculated NNT for meningitis was 23,855. Cushen 2020, evaluating children and adults, identified one brain abscess in the antibiotic treated group within 90 days of an AOM episode and no outcomes in the watchful waiting group. One additional study reported no observed cases of meningitis in 178 treated and 197 control cases [46].

Only one study assessed systemic infections and found two cases of pneumococcal bacteremia and pneumonia out of 214 subjects in the placebo group [41]. No information on pneumococcal vaccination status was available for these children.

#### Non-serious infectious complications

Three RCTs reported results for manifestation of contralateral otitis media among unilateral cases and reported a protective effect of antibiotics (RR 0.37, 0.23–0.62, Fig 2B), with an NNT of 12 [37, 50, 53]. The time frame for the development of this outcome ranged from 8 days [37] to 3 months [53].

Antibiotics showed a protective effect for acute, spontaneous TMP (RR 0.31, 0.15–0.67), with an NNT of 22 among 5 RCTs that assessed this outcome (Fig 2C) [37, 46, 47, 51, 53]. One additional study reported no cases TMP out of 42 subjects in both the placebo and antibiotic groups [39]. These perforations generally developed during the course of treatment. Three studies reporting on the healing of these perforations indicated that all perforations had healed by the end of the study, regardless of treatment [37, 51, 53]. A Bangladeshi birth cohort followed to age 2 found a trend toward a lower progression to chronic otorrhea (with TM perforation) among the antibiotic group (relative risk 0.49, 0.19–1.26) [52], but this used only 2 weeks of otorrhea, which has not qualified as “chronic” by most widely accepted definitions [54, 55]. No studies explicitly examined the impact of antibiotic treatment on perforation persistence.

Eleven studies reported AOM recurrence outcomes using a variety of definitions and timeframes from 21 days [47] to 4 years [14, 15, 38, 40, 44, 46, 47, 49, 50, 53, 56]. Antibiotics did not provide protection against AOM recurrence (RR 1.03, 0.87–1.22, Fig 2D), and several studies suggested a significant benefit of watchful waiting. To explore reasons for the heterogeneous results, we further divided recurrence into short (within less than 3 months) and long-term (within 3 or more months), which was based on the delineation of “long-term” outcomes by two studies [46, 57]. Antibiotics did not provide protection against short term AOM recurrence (RR 0.91, 0.80–1.05) as assessed by 8 RCTs [15, 38, 40, 46, 47, 50, 53, 56] or long-term AOM recurrence (RR 1.45, 0.92–2.30) as reported by 3 RCTs and 1 observational study [14, 40, 44, 49]. One prospective birth cohort study also reported results on the risk of developing recurrent AOM, defined as ≥3 episodes in 6 months or ≥4 episodes in one year. Among patients followed from birth until age 4, 12.3% treated with antibiotics and 16.7% of children treated without antibiotics developed recurrent AOM with a non-significant risk ratio of 0.79 (0.57–1.11) [44].

#### Antibiotic adverse effects

**Fig 3:**
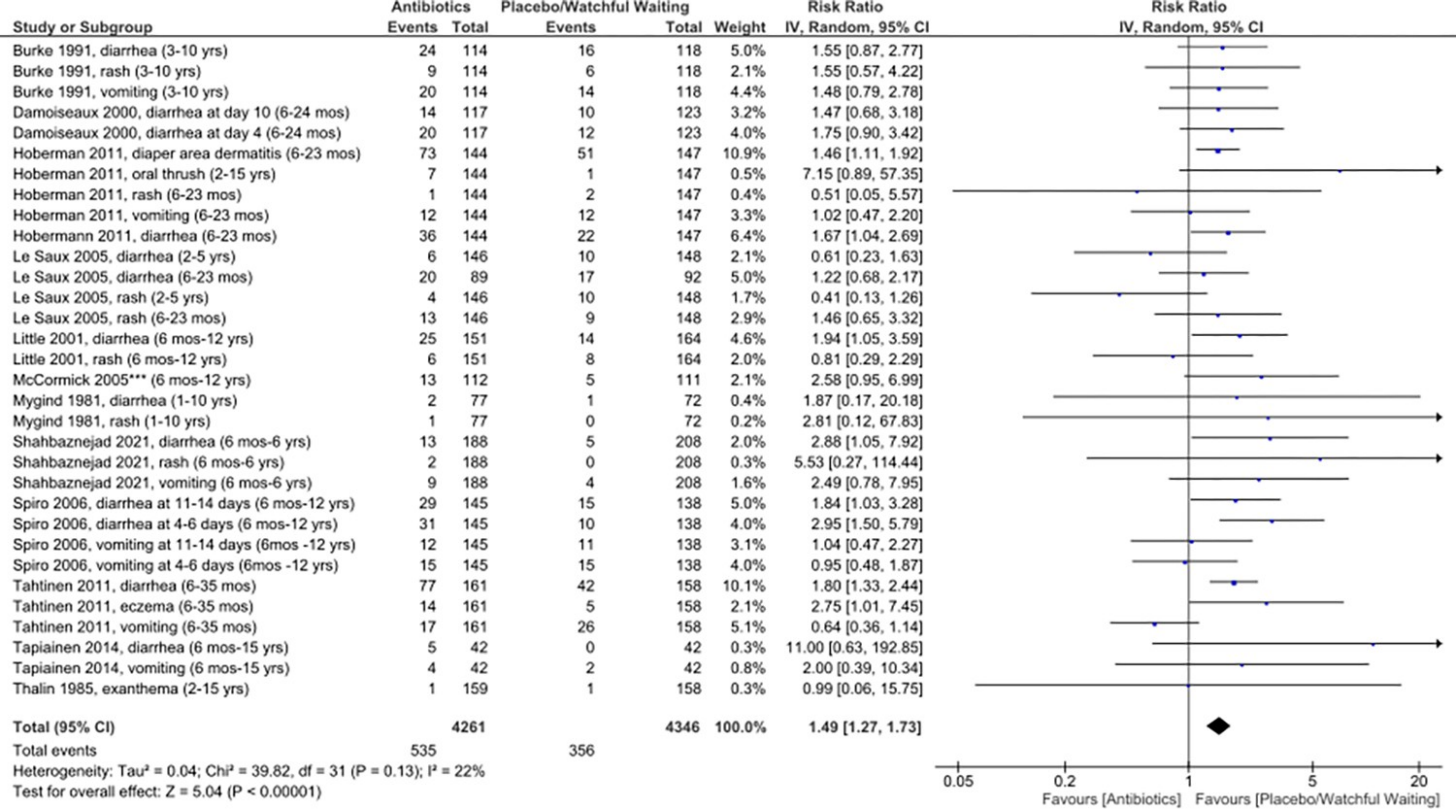
Risk ratios for antibiotic adverse effects following AOM in children treated versus not treated with antibiotics.

Twelve RCTs reported outcomes related to antibiotic adverse effects [15, 37, 39, 40, 43, 45–48, 50, 53, 58]. Adverse effects that were reported by enough studies to allow for data pooling included diarrhea, rash, and vomiting. Overall, antibiotics increased the risk of any adverse effect (RR 1.49, 95% CI 1.27–1.73), with a number needed to harm (NNH) of 23. Antibiotics increased the risk of diarrhea (RR 1.73, 95% CI 1.46–2.05, NNH 12), but did not significantly increase the risk of rash (RR 1.34, 95% CI 0.99–1.83) or vomiting (RR 1.06, 95% CI 0.78–1.44, Fig 3).

#### Sensitivity analysis

A sensitivity analysis which excluded studies of poor quality yielded similar results for all outcomes, except for TMP, where the results became non-significant changing from RR 0.31 (0.15–0.67) to 0.45 (0.19–1.019, S4 Table). The second sensitivity analysis that removed the 6 studies published prior to 2000 had similar findings (S4 Table).

## Discussion

The routine use of antibiotics to treat AOM has been in question for over 60 years [49]. Clinicians continue to prescribe antibiotics in the majority of children with AOM although watchful waiting has been explicitly adopted as an option in clinical guidelines [18, 59–61]. One common underlying reason for this practice is the fear of serious infectious complications if antibiotics are withheld [22, 24, 25]. Previous studies addressing the risk benefit, including meta-analyses that have pooled these studies, have not been able to offer conclusive evidence on the benefits of antibiotics in regard to such serious complications. Our study is the first to include observational studies to examine the impact on AOM complications, dramatically increasing sample size, thereby potentially maximizing power and follow-up time to assess results for several rare adverse outcomes.

Our study shows that the routine, prompt use of antibiotics to treat AOM in children is associated with a reduction in the risk of mastoiditis, affecting 0.017% of children with AOM with a corresponding NNT of more than 5000 patients. Other severe outcomes such as meningitis or sepsis remained too rare to support conclusive assessments. Additional positive effects of antibiotics included a reduction in the risk of contralateral AOM. In our sensitivity analysis, we found a non-significant decrease in the risk of TMP. Our findings are consistent with previous meta-analyses regarding development of several non-severe infectious complications and antibiotic adverse effects but expanded results to mastoiditis and other complications [8–10, 29].

The sum of available evidence supports a beneficial effect of antibiotics in the prevention of certain complications. However, the large NNT to avoid serious complications dwarfs the frequency of antibiotic adverse effects with an NNH of 23. To protect one additional child against the development of mastoiditis, 256 children will endure antibiotic side effects. This analysis does not consider rare, but possible life-threatening adverse effects such as anaphylaxis and Stevens-Johnson syndrome, which were not captured in the included studies, but should be considered in risk benefit decisions [62]. Antibiotic therapy may also have more insidious consequences that were not considered in this analysis, including long-term effects on the immune system [16, 63–68], and development of diabetes mellitus [69] or asthma [70]. Further research is needed to clarify the full impact on other serious complications for which we could not derive conclusive evidence.

On a broader scale, routine use of antibiotics for all children with AOM has been shown to promote the development of bacterial antibiotic resistance [71, 72]. Even isolated courses of antibiotics may lead to superinfection with resistant pneumococcus, confounding treatment of AOM and its complications [73]. The emergence of resistant pneumococcus, in turn, has been linked to a rise in the incidence of acute mastoiditis [74]. Thus, the described benefits of antibiotics may wane over time with continued widespread use. It bears mentioning that some of the studies included in our analysis predated the emergence of resistant pneumococcus and the implementation of pneumococcal vaccines, thereby introducing significant uncertainty into the protective benefit of first-line antibiotics.

The large NNT to prevent serious complications, the low NNH, and the public health impact of wide-spread antibiotic use, call into question the routine, immediate antibiotic therapy for pediatric AOM. Ideally, antibiotics would be channeled to children who may benefit to optimize the individual and public health impact of watchful waiting for those who may not. Improved understanding of risk factors for the development of AOM complications would be critical to operationalize this approach. Until these risks are more clearly understood, optimal prescribing must rely on best practices and clinical judgment. Adherence to antibiotic prescribing best practices has been shown to lead to both significant reductions in antibiotic use for childhood AOM as well as decreasing rates of acute mastoiditis [75].

We acknowledge several limitations of our meta-analysis. First, data were insufficient to draw conclusions in several areas. We intended to stratify analyses for patients 0 to 6 months and 6 months to 2 years old, but data limitations precluded this. The available data were also insufficient to draw conclusions for recurrent AOM, CSOM, hearing loss, facial palsy, intracranial complications, and systemic infectious complications. Further, several outcomes we aimed to assess were either not included or not observed in any of the studies, including cholesteatoma, tympanosclerosis, speech delay, need for tympanostomy tubes, subdural empyema, and extradural abscess. Only 7 studies reported pneumococcal vaccination rates, so the impact of vaccination on the development of these outcomes was not assessed. However, recent studies have shown that while pneumococcal vaccines have decreased the number of AOM episodes, the number of otitis-prone children [76], and overall rates of invasive pneumococcal disease [77, 78], the impact on acute mastoiditis is less clear, with one recent study showing an increase in acute mastoiditis by other Gram positive bacteria [79].

Second, inclusion of observational studies provides the sample size needed to study rare outcomes—lacking in RCTs—but brings limitations. Relying on the use of diagnostic and procedural codes for AOM could lead to the inclusion of other, more indolent conditions, such as otitis media with effusion, viral upper respiratory tract infections, and red eardrums from crying. As such non-suppurative conditions are not associated with serious infectious complications, their inclusion would bias against a protective effect of antibiotics. However, reported incidence rates reflect the risk that clinicians could expect when treating conditions they believe were AOM. Secondly, identification of outcomes also relies on the use of diagnostic and procedural codes, using criteria as determined by the study investigators (i.e., not validated). The criteria for acute mastoiditis in RCTs and observational studies were loose, including chronic and non-specific mastoid disease, considered manifestations as late as 3 months from the AOM, and did not uniformly require care for acute mastoiditis (e.g., admission, parenteral antibiotics, cortical mastoidectomy). This could introduce misclassification bias which could underestimate the protective effect of antibiotics for AOM management. Observational studies also have the potential for confounding since randomization of the study groups has not occurred and complete adjustment is not always possible, although we did not notice substantial difference in effect estimates when compared to RCTs, alone. Reliance on pharmacy records of antibiotic prescriptions and assumption that patients are administered the antibiotics may also introduce bias. Some patients may have been given the antibiotic during the clinic visit, which may not have been captured. In this vein, observational studies may have missed outcomes in patients who did not seek care, although similar limitations may apply to RCTs, as well.

Finally, in this meta-analysis, we included watchful waiting, placebo, and no antibiotic treatment as our comparators. Although watchful waiting cohorts may have received antibiotics eventually if warranted by their clinical condition, combining these control groups allowed us to adequately study whether a decrease in immediate antibiotic use would increase the risk of complications in children with AOM. The placebo cohorts in RCTs likely also followed current guidelines and included rescue antibiotics, if necessary, which is tantamount to watchful waiting.

## Conclusions

Prompt antibiotic therapy for AOM reduces the risk for AM, transient TMP, and contralateral AOM, while increasing the risk for adverse antibiotic effects. The NNT to avoid serious complications is very high, and the NNH is relatively low. Despite the inclusion of large population-based observational studies, sample size constraints preclude the assessment of the most serious infectious complications. Validation of measures to reliably detect AOM complications is needed to leverage the power of observational studies using large real-world datasets. Use of such real-world data could then support identification of risk factors for the development of AOM complications to channel antibiotics to children that may truly benefit and to optimize the individual and public health impact of watchful waiting.

## Supporting information

S1 Checklist PRISMA 2020 for abstracts checklist. (DOCX)

S2 Checklist PRISMA 2020 checklist. (DOCX)

S1 Table Search strategy for complications of AOM. (PDF)

S2 Table Patient baseline characteristics for included studies. (PDF)

S3 Table AOM recurrence outcome definitions and timeframes for included studies. (PDF)

S4 Table Risk ratios for primary and sensitivity analyses. (PDF)

S1 Fig Risk of bias in randomized trials (RoB 2) assessment summary. (TIF)

S2 Fig Risk of bias in non-randomized studies of interventions (ROBINS-I) assessment summary. (TIF)

S3 Fig Funnel plot for studies evaluating mastoiditis. (TIF)

S4 Fig Funnel plots for studies evaluating non-serious infectious complications. (PDF)

S5 Fig Funnel plots for studies evaluating antibiotic adverse effects. (PDF)

S6 Fig Risk ratios for contralateral AOM following AOM in children treated versus not treated with antibiotics. (TIF)

S7 Fig Risk ratios for diarrhea following AOM in children treated versus not treated with antibiotics. (TIF)

S8 Fig Risk ratios for rash following AOM in children treated versus not treated with antibiotics. (TIF)

S9 Fig Risk ratios for vomiting following AOM in children treated versus not treated with antibiotics. (TIF)
