# Supplementary material for: Antibiotic treatment to prevent pediatric acute otitis media infectious complications: A meta-analysis
Source: PLoS One. 2024 Jun 17;19(6):e0304742. doi: 10.1371/journal.pone.0304742 (PMC11182555; doi:10.1371/journal.pone.0304742)
Supplement: S1 Table — (PDF) [file pone.0304742.s003.pdf]

## S1 Table. Search strategy for complications of AOM

| Database | Search Strategies |
|---|---|
| | (“Acute Otitis Media”) **AND** (“Complications”) **AND** (“Antibiotic Treatment”) **AND** (“Pediatric Population”) **AND** (“Study Type”) |
| **PubMed/ MEDLINE** | ("Otitis Media"[MeSH] OR "acute otitis"[tiab] OR "acute otitis media"[tiab] OR "middle ear infection"[tiab] OR "middle ear inflammat*"[tiab] OR "otitis media"[tiab])<br>**AND**<br>("Hearing Loss"[Mesh] OR "Mastoiditis"[Mesh] OR "Cholesteatoma"[Mesh] OR "Myringosclerosis"[Mesh] OR "Middle Ear Ventilation"[Mesh] OR "Meningitis"[Mesh] OR "Brain Abscess"[Mesh] OR "Empyema, Subdural"[Mesh] OR "Epidural Abscess"[Mesh] OR "Sepsis"[Mesh] OR "Death"[Mesh] OR "Language Development Disorders"[Mesh] OR "Recurrence"[Mesh] OR "Long Term Adverse Effects"[Mesh] OR "Drug-Related Side Effects and Adverse Reactions"[Mesh] OR "Otitis Media/complications"[Mesh] OR "Anti-Bacterial Agents/adverse effects"[Mesh] OR "Intratemporal complication*"[tiab] OR "hearing loss"[tiab] OR "hearing impairment"[tiab] OR "hearing difficulty"[tiab] OR "deafness"[tiab] OR "spontaneous tympanic membrane perforation"[tiab] OR "mastoiditis"[tiab] OR "cholesteatoma"[tiab] OR "chronic suppurative otitis media"[tiab] OR "tympanosclerosis"[tiab] OR "Tympanostomy tube*"[tiab] OR "contralateral otitis media"[tiab] OR "Intracranial complication*"[tiab] OR "meningitis"[tiab] OR "brain abscess"[tiab] OR "subdural empyema"[tiab] OR "extradural abscess"[tiab] OR "systemic complication*"[tiab] OR "sepsis"[tiab] OR "death"[tiab] OR "downstream complication*"[tiab] OR "speech delay"[tiab] OR recurr*[tiab] OR "adverse event*"[tiab] OR "adverse effect*"[tiab] OR "adverse reaction"[tiab] OR "adverse drug"[tiab] OR "Treatment Failure"[MeSH] OR "treatment failure"[tiab]<br>OR “long-term sequelae”[tiab] OR “longterm sequelae”[tiab])<br>**AND**<br>("anti-bacterial agents"[MeSH] OR "anti-bacterial agents" [Pharmacological Action] OR "antibiotic"[tiab] OR "antibiotics"[tiab])<br>**AND**<br>("Child" [Mesh] OR "Child, Preschool" [Mesh] OR "Pediatrics" [MeSH] OR child* [tiab] OR Pediatric* [tiab] OR toddler* [tiab] OR "youth" [tiab])<br>**AND**<br>("Comparative Study" [Publication Type] OR "Retrospective Studies"[Mesh] OR "Cohort Studies"[Mesh] OR “comparative”[tiab] OR “control”[tiab] OR “controlled”[tiab] OR “cohort”[tiab] OR “observation*”[tiab] OR “retrospective”[tiab]) |
| **Embase** | ('acute otitis media'/exp OR 'acute otitis':ti,ab OR 'acute otitis media':ti,ab OR 'otitis media acuta':ti,ab OR 'otitis media'/exp OR 'false latent otitis':ti,ab OR 'middle ear infection':ti,ab OR 'middle ear infections':ti,ab OR 'middle ear inflammation':ti,ab OR 'middle ear inflammatory disease':ti,ab OR 'middle ear inflammatory pathology':ti,ab OR 'middle ear inflammatory process':ti,ab OR 'otitis media':ti,ab)<br>**AND**<br>('adverse event'/exp OR 'adverse effect':ti,ab OR 'adverse effects':ti,ab OR 'adverse event':ti,ab OR 'adverse events':ti,ab OR 'adverse reaction':ti,ab OR 'adverse drug reaction'/exp OR 'adverse drug effect':ti,ab OR 'adverse drug event':ti,ab OR 'adverse drug reaction':ti,ab OR 'adverse reaction, drug':ti,ab OR 'drug adverse effect':ti,ab OR 'drug adverse reaction':ti,ab OR 'drug reaction, adverse':ti,ab OR 'drug side effect':ti,ab OR 'drug-related side effects and adverse reactions':ti,ab OR 'long term adverse effects':ti,ab OR 'metabolic side effects of drugs and substances':ti,ab OR 'complication'/exp OR 'complication':ti,ab OR 'complications':ti,ab OR 'hearing impairment'/exp OR 'auditory defect':ti,ab OR 'deaf':ti,ab OR 'deafness':ti,ab OR 'hearing damage':ti,ab OR 'hearing defect':ti,ab OR 'hearing difficulty':ti,ab OR 'hearing impairment':ti,ab OR 'hearing loss':ti,ab OR 'hypacusia':ti,ab OR 'hypacusis':ti,ab OR 'hypoacousia':ti,ab OR 'hypoacusis':ti,ab OR 'impaired hearing':ti,ab OR 'spontaneous tympanic membrane perforation':ti,ab OR |

| | |
|---|---|
| | 'mastoiditis'/exp OR 'acute mastoiditis':ti,ab OR 'infection, mastoid':ti,ab OR 'inflammation, mastoid':ti,ab OR 'mastoid infection':ti,ab OR 'mastoid inflammation':ti,ab OR 'mastoiditis':ti,ab OR 'mastoiditis, acute':ti,ab OR 'cholesteatoma'/exp OR 'cholesteatoma':ti,ab OR 'cholesteatoma, middle ear':ti,ab OR 'chronic suppurative otitis media'/exp OR 'csom (chronic suppurative otitis media)':ti,ab OR 'chronic purulent otitis media':ti,ab OR 'chronic suppurative otitis media':ti,ab OR 'tympanosclerosis'/exp OR tympanosclerosis:ti,ab OR 'tympanostomy tube'/exp OR 'activent':ti,ab OR 'armstrong (tympanostomy tube)':ti,ab OR 'armstrong v grommets':ti,ab OR 'goode t-tube':ti,ab OR 'paparella i':ti,ab OR 'paparella ii':ti,ab OR 'soileau tytan':ti,ab OR 'tula system (tube delivery system)':ti,ab OR 'ear ventilation tube':ti,ab OR 'grommet':ti,ab OR 'microgel (tympanostomy tube)':ti,ab OR 'middle ear tube':ti,ab OR 'tympanic ventilation tube':ti,ab OR 'tympanic ventilation tube (physical object)':ti,ab OR 'tympanostomy tube':ti,ab OR 'ventilating tube, ear':ti,ab OR 'ventilation tube (ear)':ti,ab OR 'contralateral otitis media':ti,ab OR 'intracranial complication':ti,ab OR 'meningitis'/exp OR 'leptospirotic meningitis':ti,ab OR 'meningeal inflammation':ti,ab OR 'meningitis':ti,ab OR 'meningitis, recurrent':ti,ab OR 'perimeningeal infections':ti,ab OR 'recurrent meningitis':ti,ab OR 'brain abscess'/exp OR 'abscess, brain':ti,ab OR 'brain abscess':ti,ab OR 'cerebral abscess':ti,ab OR 'cerebrum abscess':ti,ab OR 'encephalopyosis':ti,ab OR 'intracranial abscess':ti,ab OR 'subdural empyema'/exp OR 'empyema, subdural':ti,ab OR 'subdural abscess':ti,ab OR 'subdural abscesses':ti,ab OR 'subdural empyema':ti,ab OR 'subdural empyemas':ti,ab OR 'epidural abscess'/exp OR 'abscess, epidural':ti,ab OR 'abscess, extradural':ti,ab OR 'epidural abscess':ti,ab OR 'extradural abscess':ti,ab OR 'systemic complication':ti,ab OR 'systemic complications':ti,ab OR 'sepsis'/exp OR 'death'/exp OR 'death':ti,ab OR 'mors':ti,ab OR 'downstream complication':ti,ab OR 'downstream complications':ti,ab OR 'speech delay'/exp OR 'alalia':ti,ab OR 'delayed speech':ti,ab OR 'delayed speech development':ti,ab OR 'late talker':ti,ab OR 'speech delay':ti,ab OR 'recurrence risk'/exp OR 'recidivation risk':ti,ab OR 'recidivism risk':ti,ab OR 'recurrence rate':ti,ab OR 'recurrence risk':ti,ab OR 'relapse rate':ti,ab OR 'risk recidivism':ti,ab OR 'risk, recurrence':ti,ab OR 'long-term sequelae':ti,ab OR 'longterm sequelae':ti,ab)<br>**AND**<br>('antibiotic agent'/exp OR 'antibiotics':ti,ab OR 'antibiotic':ti,ab OR 'anti-bacterial':ti,ab)<br>**AND**<br>('pediatric'/exp OR 'pediatrics'/exp OR 'paediatric care':ti,ab OR 'paediatrics':ti,ab OR 'pediatrics':ti,ab OR 'child'/exp OR 'child':ti,ab OR 'children':ti,ab OR 'toddler'/exp OR 'toddler':ti,ab OR 'toddlers':ti,ab OR 'infant'/de OR 'infant':ti,ab OR 'baby'/exp OR 'baby')<br>**AND**<br>('retrospective study'/exp OR 'retrospective design':ti,ab OR 'retrospective panel studies':ti,ab OR 'retrospective panel study':ti,ab OR 'retrospective studies':ti,ab OR 'retrospective study':ti,ab OR 'study, retrospective':ti,ab OR 'cohort analysis'/exp OR 'analysis, cohort':ti,ab OR 'cohort analysis':ti,ab OR 'cohort studies':ti,ab OR 'cohort study':ti,ab OR 'comparative study'/exp OR 'comparative studies':ti,ab OR 'comparative study':ti,ab OR 'comparison':ti,ab OR 'control'/exp OR 'control':ti,ab OR 'controlled study'/exp OR 'control group study':ti,ab OR 'control group trial':ti,ab OR 'controlled study':ti,ab OR 'controlled trial':ti,ab OR 'observational study'/exp OR 'observation studies':ti,ab OR 'observation study':ti,ab OR 'observational studies':ti,ab OR 'observational studies as topic':ti,ab OR 'observational study':ti,ab OR 'observational study as topic':ti,ab) |
| **Cochrane Database of Systematic Reviews**<br><br>**and**<br><br>**Cochrane Central** | ("acute otitis" OR "acute otitis media" OR "middle ear infection" OR "middle ear inflammation" OR "otitis media")<br>**AND**<br>("Intratemporal complication" OR "Intratemporal complications" OR "hearing loss" OR "hearing impairment" OR "hearing difficulty" OR "deafness" OR "spontaneous tympanic membrane perforation" OR "mastoiditis" OR "cholesteatoma" OR "chronic suppurative otitis media" OR "tympanosclerosis" OR "Tympanostomy tube" OR "contralateral otitis media" OR "Intracranial complication" OR "intracranial complications" OR "meningitis" OR "brain abscess" OR "subdural empyema" OR "extradural abscess" OR "systemic complication" OR "systemic complications" OR "sepsis" OR "death" OR "downstream complication" OR |

| | |
|---|---|
| **Register of Controlled Trials** | “downstream complications” OR "speech delay" OR “recurrence” OR “recurrent” OR “recurring” OR "adverse event” OR “adverse events” OR "adverse effect" OR “adverse effects” OR "adverse reaction" OR "adverse drug" OR "treatment failure" OR “long-term sequelae” OR “longterm sequelae”)<br>**AND**<br>("anti-bacterial" OR "antibiotic" OR "antibiotics")<br>**AND**<br>(child OR children OR Pediatric OR Pediatrics OR toddler OR toddlers OR "youth")<br>**AND**<br>(“comparative” OR “control” OR “controlled” OR “cohort” OR “observation” OR “observational” OR “retrospective”) |
| **Web of Science** | ("acute otitis" OR "acute otitis media" OR "middle ear infection" OR "middle ear inflammation” OR "otitis media")<br>**AND**<br>("Intratemporal complication” OR “Intratemporal complications” OR "hearing loss" OR "hearing impairment" OR "hearing difficulty" OR "deafness" OR "spontaneous tympanic membrane perforation" OR "mastoiditis" OR "cholesteatoma" OR "chronic suppurative otitis media" OR "tympanosclerosis"  OR "Tympanostomy tube” OR "contralateral otitis media" OR "Intracranial complication” OR “intracranial complications” OR "meningitis" OR "brain abscess" OR "subdural empyema" OR "extradural abscess" OR "systemic complication” OR “systemic complications” OR "sepsis" OR "death" OR "downstream complication” OR “downstream complications” OR "speech delay" OR “recurrence” OR “recurrent” OR “recurring” OR "adverse event” OR “adverse events” OR "adverse effect" OR “adverse effects” OR "adverse reaction" OR "adverse drug" OR "treatment failure" OR “long-term sequelae” OR “longterm sequelae”)<br>**AND**<br>("anti-bacterial" OR "antibiotic" OR "antibiotics")<br>**AND**<br>(child OR children OR Pediatric OR Pediatrics OR toddler OR toddlers OR "youth")<br>**AND**<br>(“comparative” OR “control” OR “controlled” OR “cohort” OR “observation” OR “observational” OR “retrospective”) |
