# Supplementary material for: Antibiotic treatment to prevent pediatric acute otitis media infectious complications: A meta-analysis
Source: PLoS One. 2024 Jun 17;19(6):e0304742. doi: 10.1371/journal.pone.0304742 (PMC11182555; doi:10.1371/journal.pone.0304742)
Supplement: S2 Table — (PDF) [file pone.0304742.s004.pdf]

S2 Table. Patient baseline characteristics for included studies

Study	Study Groups	Sex, n (%) male	White, Non-Hispanic, n (%)	Hispanic, n (%)	Asian/ Pacific Islander, n (%)	Black, n (%)	Other, n (%)	Pneumococcal Vaccination**, n (%)	Average age in study, mean ± STD	Breastfed (y/n or average duration), n (%)	Siblings, n (%)	Smoking exposure, n (%)	Attendance at daycare or school, n (%)
**Bezakova 2009**	**Intervention**	43 (55)	-	-	-	-	-	-	13.5 months old	For > 6 months: 17 (22)	>2 children in family 19 (24)	27 (35)	19 (24)
	**Control**	45 (50)	-	-	-	-	-	-	13.1 months old	For > 6 months: 17 (19)	>2 children in family 18 (20)	24 (27)	16 (18)
**Burke 1991**	**Intervention**	59 (52)	-	-	-	-	-	-	-	-	-	-	-
	**Control**	50 (42)	-	-	-	-	-	-	-	-	-	-	-
**Cars 2017**	**Intervention**	-	-	-	-	-	-	-	-	-	-	-	-
	**Control**	-	-	-	-	-	-	-	-	-	-	-	-
**Cushen 2020**	**Intervention**	-	-	-	-	-	-	-	6 years old	-	-	-	-
	**Control**	-	-	-	-	-	-	-	6 years old	-	-	-	-
**Damoiseaux 2000**	**Intervention**	64 (54.7)	-	-	-	-	-	-	13.3 months old	For > 6 months: 21 (17.9)	>2 children in family: 30 (25.6)	46 (39.3)	28 (23.9)
	**Control**	66 (53.7)	-	-	-	-	-	-	13.3 months old	For > 6 months: 22 (17.9)	> 2 children in family: 25 (20.3)	39 (31.7)	19 (15.4)
**Hoberman 2011**	**Intervention**	75 (52)	66 (46)	-	-	62 (43)	16 (11)	144 (100)	-	-	-	-	Exposure to other children: 69 (48)
	**Control**	80 (54)	65 (44)	-	-	58 (39)	24 (16)	147 (100)	-	-	-	-	Exposure to other children: 72 (49)
**Kaleida 1991**	**Intervention**	130 (49.4)*	230 (87.5)*	-	-	-	Nonwhite: 33 (12.6)*	-	-	-	-	-	-
	**Control**	150 (54.9)*	244 (89.4)*	-	-	-	Nonwhite: 29 (10.7)*	-	-	-	-	-	-
**Laxdal 1970**	**Intervention**	-	-	-	-	-	-	-	-	-	-	-	-
	**Control**	-	-	-	-	-	-	-	-	-	-	-	-
**Le Saux 2005**	**Intervention**	129 (50)	-	-	-	-	-	0	3 years old ± 1.61	-	Number of siblings at home, median (range): 1 (0-6)	68 (26.3)	109 (42.2)

	**Control**	132 (52)	-	-	-	-	-	0	2.87 years old ± 1.59	-	Number of siblings at home, median (range): 1 (0-4)	67 (26.4)	121 (47.6)
**Little 2001***	**Intervention**	79 (52)*	-	-	-	-	-	-	-	-	-	-	-
	**Control**	77 (47)*	-	-	-	-	-	-	-	-	-	-	-
**McCormick 2005**	**Intervention**	54 (48)	34 (30)	42 (38)	1 (1)	24 (21)	11 (10)	17 (15)	-	Mean duration: 2.6 months ± 3.7	-	36 (32)	52 (46.4)
	**Control**	58 (52)	27 (24)	37 (33)	6 (5)	26 (24)	15 (14)	20 (18)	-	Mean duration: 2.6 months ± 4.8	-	29 (26)	52 (46.8)
**Molder 2016**	**Intervention**	276 (53.9)	-	-	-	-	-	-	-	395 (77.1)	355 (69.3)	During first year of life: 94 (18.4)	During first year of life: 429 (83.8)
	**Control**	166 (49.4)	-	-	-	-	-	-	-	277 (82.4)	196 (58.3)	During first year of life: 75 (22.3)	During first year of life: 279 (83.0)
**Mygind 1981**	**Intervention**	36 (50)	-	-	-	-	-	-	3.7 years old	-	-	-	59 (81)
	**Control**	38 (49.3)	-	-	-	-	-	-	4.1 years old	-	-	-	66 (85)
**Petersen 2007**	**Intervention**	-	-	-	-	-	-	-	-	-	-	-	-
	**Control**	-	-	-	-	-	-	-	-	-	-	-	-
**Roy 2012**	**Intervention**	143 (56.7)	-	-	-	-	-	-	-	-	-	-	-
	**Control**		-	-	-	-	-	-	-	-	-	-	-
**Ruohola 2018***	**Intervention**	92 (57)	-	-	-	-	-	3 (1.9)	16 months old	Mean duration: 7 months	89 (55)	57 (36)	87 (54)
	**Control**	90 (57)	-	-	-	-	-	4 (2.5)	16 months old	Mean duration: 7 months	93 (59)	48 (30)	86 (54)
**Shahbaznejad 2021**	**Intervention**	104 (55)	-	-	-	-	-	3 (1.6)	29.05 months old ± 16.6	Exclusive breastfeeding: 128 (68)	-	30 (16)	48 (26)
	**Control**	95 (46)	-	-	-	-	-	3 (1.4)	28.88 months old ± 15.9	Exclusive breastfeeding: 136 (66)	-	37 (18)	57 (27)
**Spiro 2006**	**Intervention**	76 (52)	18 (12)	61 (42)	-	62 (43)	4 (3)	-	3.2 years old	-	-	51 (38)	74 (56)
	**Control**	79 (57)	13 (9)	65 (47)	-	49 (36)	11 (8)	-	3.6 years old	-	-	44 (33)	71 (54)

**Tahtinen 2011***	**Intervention**	92 (57)	-	-	-	-	-	3 (1.9)	16 months old	Mean duration: 7 months	89 (55)	57 (36)	87 (54)
	**Control**	90 (57)	-	-	-	-	-	4 (2.5)	16 months old	Mean duration: 7 months	93 (59)	48 (30)	86 (54)
**Tahtinen 2012***	**Intervention**	92 (57)	-	-	-	-	-	3 (1.9)	16 months old	155 (96.3)	89 (55)	57 (36)	87 (54)
	**Control**	27 (51)	-	-	-	-	-	3 (6)	-	51 (96.2)	-	13 (25)	36 (68)
**Tapiainen 2014**	**Intervention**	19 (45)	-	-	-	-	-	3 (7)	4.5 years old ± 3.1	-	-	11 (26)	35 (83.3)
	**Control**	17 (41)	-	-	-	-	-	2 (5)	4.3 years old ± 2.8	-	-	10 (24)	37 (88.1)
**Thalin 1985**	**Intervention**	-	-	-	-	-	-	-	-	-	-	-	-
	**Control**	-	-	-	-	-	-	-	-	-	-	-	-
**Thompson 2009**	**Intervention**	604, 113 (51.1)	-	-	-	-	-	-	5.4 years old	-	-	-	-
	**Control**		-	-	-	-	-	-		-	-	-	-
**van Buchem 1981**	**Intervention**	-	-	-	-	-	-	-	-	-	-	-	-
	**Control**	-	-	-	-	-	-	-	-	-	-	-	-

* Ruohola 2018 and Tahtinen 2011 include the same cohorts of patients. Tahtinen 2011 and Tahtinen 2012 include the same cohort of patients treated with immediate antibiotics.
