# Supplementary material for: Antibiotic treatment to prevent pediatric acute otitis media infectious complications: A meta-analysis
Source: PLoS One. 2024 Jun 17;19(6):e0304742. doi: 10.1371/journal.pone.0304742 (PMC11182555; doi:10.1371/journal.pone.0304742)
Supplement: S3 Table — (PDF) [file pone.0304742.s005.pdf]

## S3 Table. AOM recurrence outcome definitions and timeframes for included studies

| Study | Recurrence Definition | Recurrence Timeframe |
|---|---|---|
| **Bezakova 2009** | Parent-reported AOM recurrence recorded in questionnaire | 6 months to 3.5 years |
| **Hoberman 2011** | The reappearance of acute otitis media in a patient previous classified as clinical success (days 10-12) at visit on days 21-25 | 21-25 days |
| **Kaleida 1991** | Development of AOM 15 or more days after initiation of treatment for a preceding episode | 2-6 weeks |
| **Laxdal 1970** | No definition provided, termed "relapses" | 1-33 months (mean: 16 months) |
| **Le Saux 2005** | No definition provided, termed "recurrence" | 1 or 3 months |
| **McCormick 2005** | Returning to office with acute symptoms on days 13-30 with acute ear symptoms, abnormal tympanic membrane, and an AOM-Si score higher than at enrollment | 30 days |
| **Molder 2016** | New episode defined as 28 days since prior AOM-related consultation | 4 years |
| **Mygind 1981** | Not provided | 3 months |
| **Ruohola 2018** | Recurrence assessed during scheduled visits | 90 days +/- 10 days |
| **Shahbaznejad 2021** | Patient reported AOM symptoms recurrence | 3 months |
| **Thalin 1985** | Recurrence of AOM after the end of medical treatment and before day 30 | 30 days |
