# Supplementary material for: Antibiotic treatment to prevent pediatric acute otitis media infectious complications: A meta-analysis
Source: PLoS One. 2024 Jun 17;19(6):e0304742. doi: 10.1371/journal.pone.0304742 (PMC11182555; doi:10.1371/journal.pone.0304742)
Supplement: S4 Table — (PDF) [file pone.0304742.s006.pdf]

S4 Table. Risk ratios for primary and sensitivity analyses

Outcome	Primary Analysis RR (95% CI)	Sensitivity Analysis with poor-quality studies removed RR (95% CI)	Sensitivity Analysis with studies published prior to 2000 removed RR (95% CI)
Any antibiotic adverse effect	1.49 (1.27-1.73)	1.50 (1.32-1.70)	1.50 (1.31-1.71)
Contralateral otitis media	0.37 (0.23-0.62)	0.43 (0.24-0.76)	0.44 (0.24-0.81)
Recurrence	1.03 (0.87-1.22)	0.94 (0.87-1.02)	0.90 (0.78-1.03)
Short term recurrence	0.91 (.80-1.04)	0.90 (0.79-1.03)	0.84 (0.67-1.05)
Long-term recurrence	1.45 (0.92-2.30)	0.97(0.87-1.07)	0.97 (0.87-1.07)
Diarrhea	1.73 (1.46-2.05)	1.71 (1.43-2.04)	1.72 (1.38-2.14)
Mastoiditis	0.48 (0.40-0.59)	No studies excluded	No studies excluded
Rash	1.34 (0.99-1.83)	1.33 (.93-1.89)	1.25 (0.79-1.97)
Tympanic membrane perforation	0.31 (0.15-0.67)	0.45 (0.19-1.09)	0.36 (0.07-1.83)
Vomiting	1.06 (0.78-1.44)	No studies excluded	0.97 (0.69-1.35)
