# Supplementary figures and images for: Antibiotic treatment to prevent pediatric acute otitis media infectious complications: A meta-analysis
Source: PLoS One. 2024 Jun 17;19(6):e0304742. doi: 10.1371/journal.pone.0304742 (PMC11182555; doi:10.1371/journal.pone.0304742)

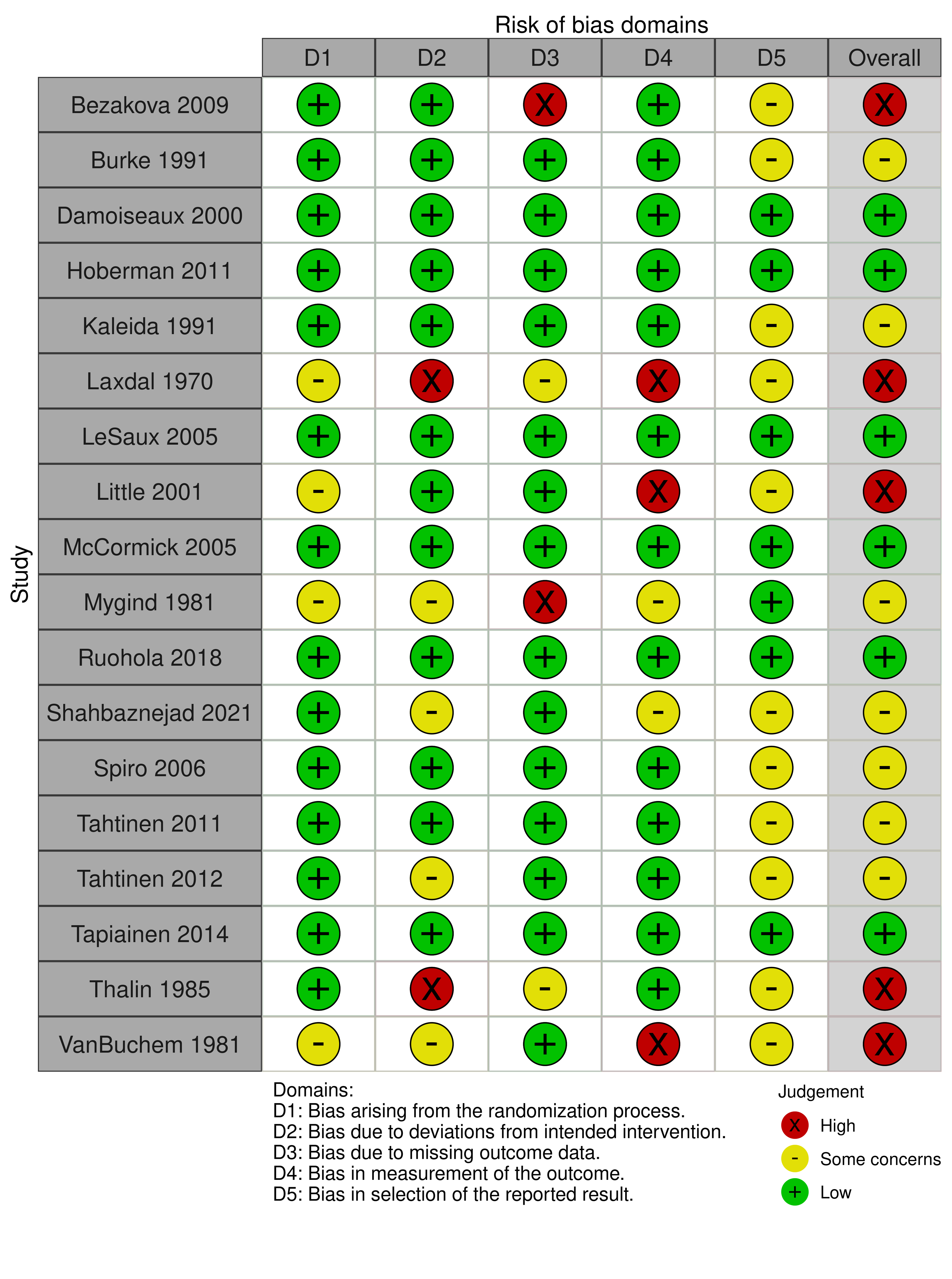

Supplement: S1 Fig — (TIF) [file pone.0304742.s007.tif]

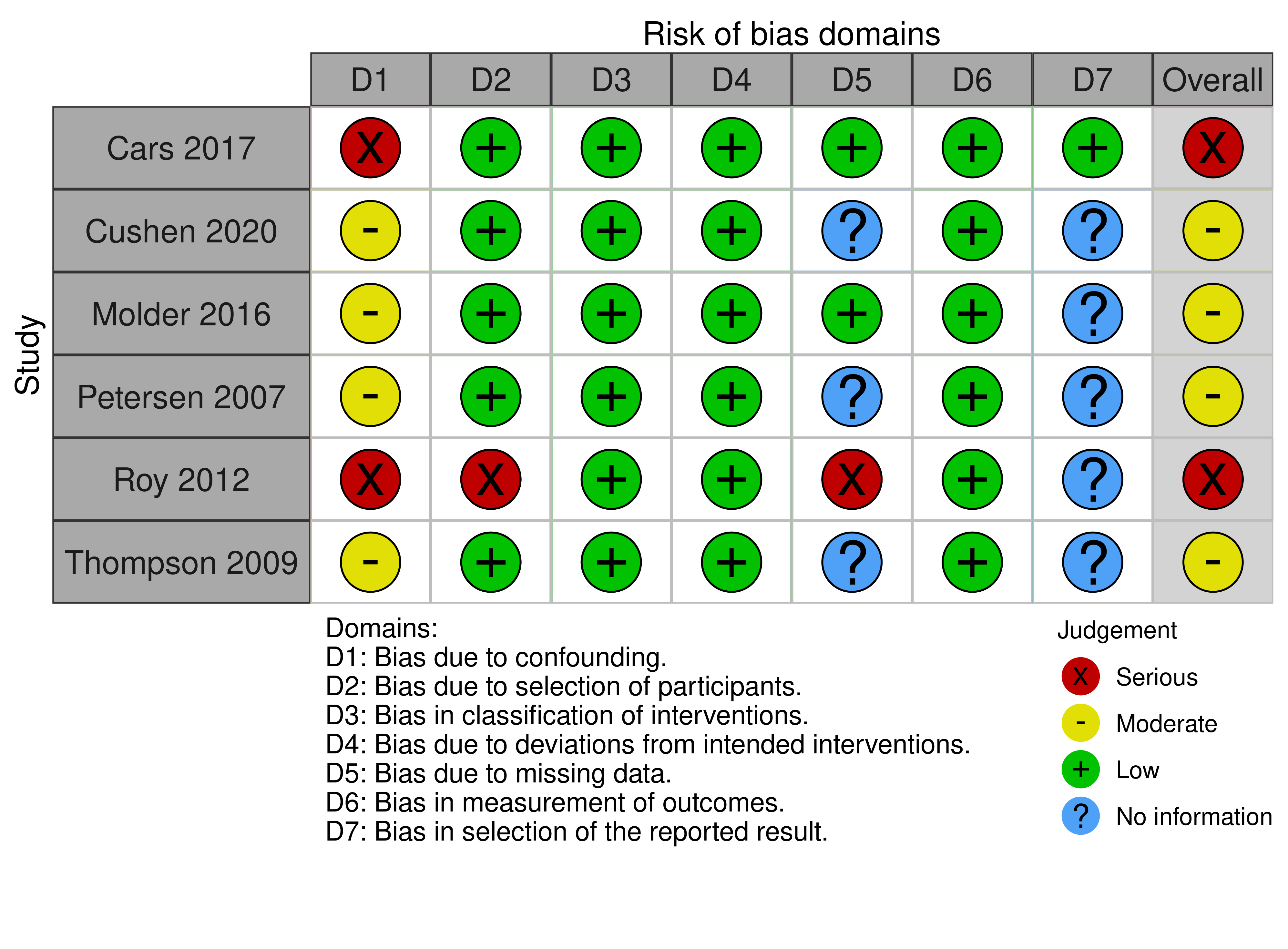

Supplement: S2 Fig — (TIF) [file pone.0304742.s008.tif]

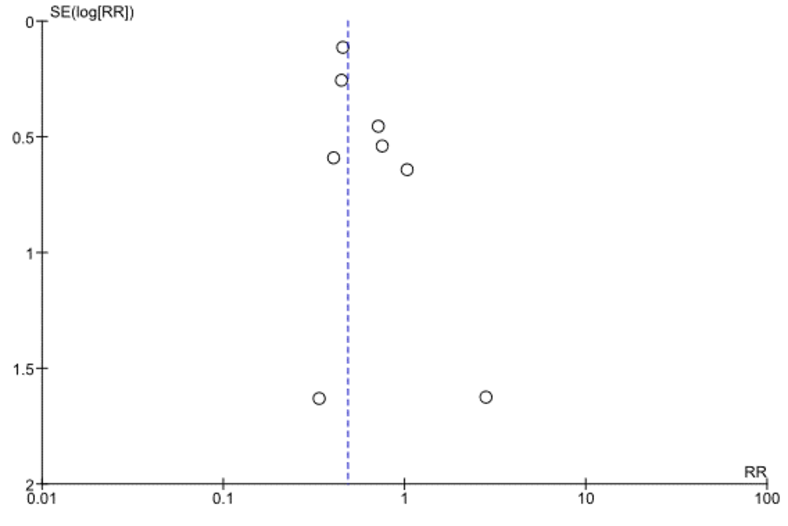

Supplement: S3 Fig — (TIF) [file pone.0304742.s009.tif]

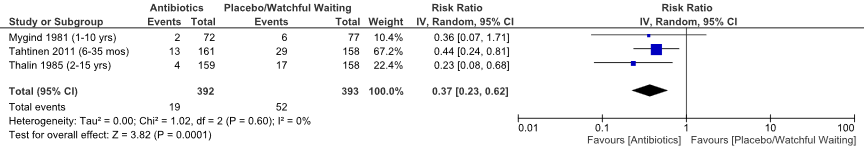

Supplement: S6 Fig — (TIF) [file pone.0304742.s012.tif]

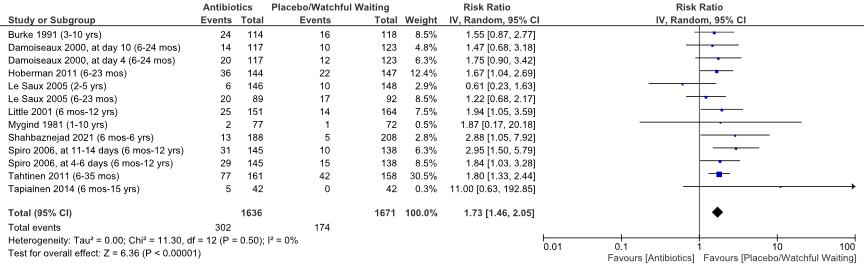

Supplement: S7 Fig — (TIF) [file pone.0304742.s013.tif]

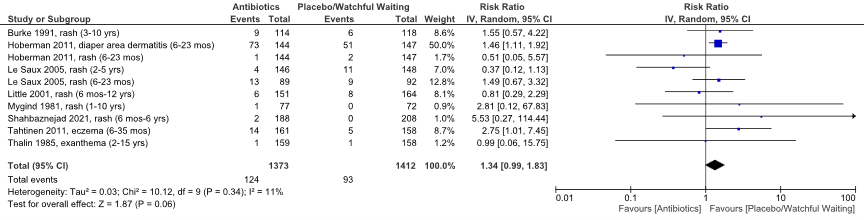

Supplement: S8 Fig — (TIF) [file pone.0304742.s014.tif]

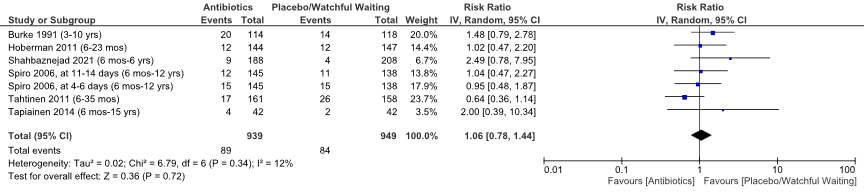

Supplement: S9 Fig — (TIF) [file pone.0304742.s015.tif]
