# Supplementary material for: Antibiotic treatment to prevent pediatric acute otitis media infectious complications: A meta-analysis
Source: PLoS One. 2024 Jun 17;19(6):e0304742. doi: 10.1371/journal.pone.0304742 (PMC11182555; doi:10.1371/journal.pone.0304742)
Supplement: S4 Fig — (PDF) [file pone.0304742.s010.pdf]

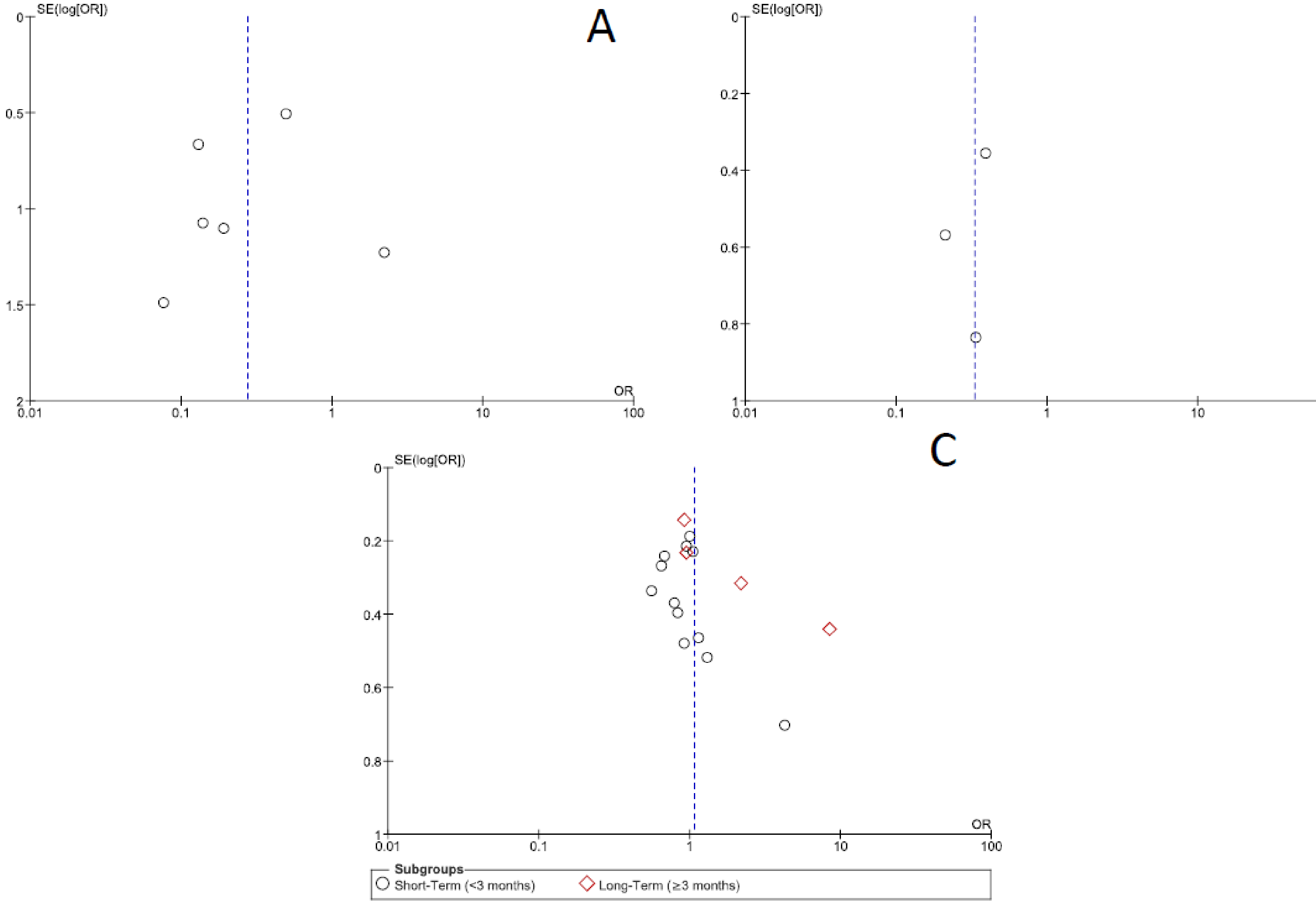

S4 Fig. Funnel plots for studies evaluating non-serious infectious complications

S4a corresponds to tympanic membrane perforation, S4b to contralateral AOM, S4c to AOM recurrence
