# Supplementary material for: Antibiotic treatment to prevent pediatric acute otitis media infectious complications: A meta-analysis
Source: PLoS One. 2024 Jun 17;19(6):e0304742. doi: 10.1371/journal.pone.0304742 (PMC11182555; doi:10.1371/journal.pone.0304742)
Supplement: S5 Fig — (PDF) [file pone.0304742.s011.pdf]

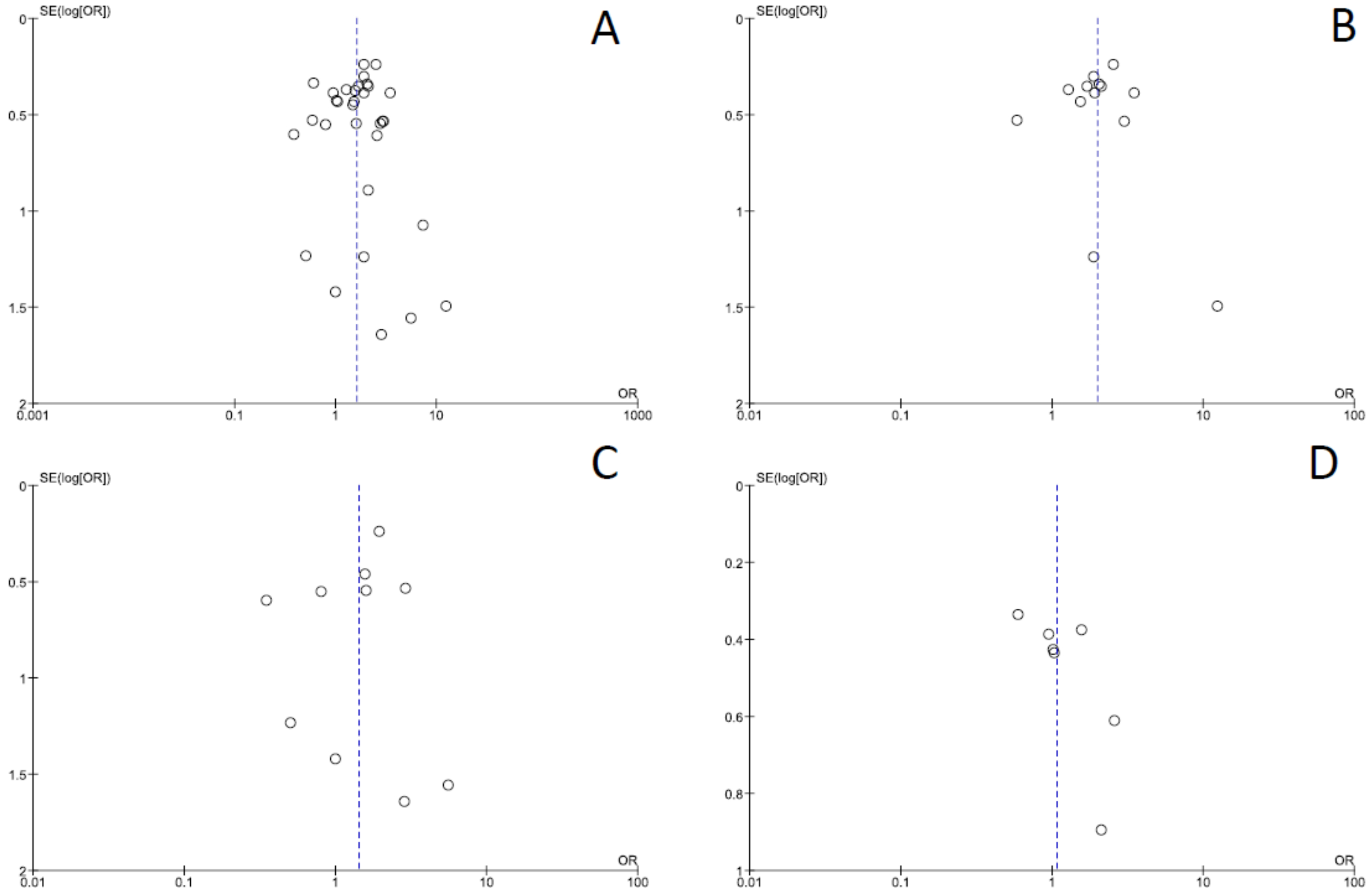

S5 Fig. Funnel plots for studies evaluating antibiotic adverse effects

S5a shows publication bias for all antibiotic adverse effects, S5b corresponds to diarrhea, S5c to rash, S5d to vomiting
